# Outcomes After Implementation of a Benzodiazepine-Sparing Alcohol Withdrawal Order Set in an Integrated Health Care System

**DOI:** 10.1001/jamanetworkopen.2022.0158

**Published:** 2022-02-22

**Authors:** Joshua T. Smith, Mary Sage, Herb Szeto, Laura C. Myers, Yun Lu, Adriana Martinez, Patricia Kipnis, Vincent X. Liu

**Affiliations:** 1 Division of Research, Kaiser Permanente Northern California, Oakland; 2 Consultation-Liaison Psychiatry Service, Walnut Creek Medical Center, Kaiser Permanente Northern California, Walnut Creek; 3 Hospital Based Specialist Service, Redwood City Medical Center, Kaiser Permanente Northern California, Redwood City; 4 School of Medicine, University of California, Davis, Sacramento

## Abstract

**Importance:**

Alcohol withdrawal syndrome (AWS) is a common inpatient diagnosis managed primarily with benzodiazepines. Concerns about the adverse effects associated with benzodiazepines have spurred interest in using benzodiazepine-sparing treatments.

**Objective:**

To evaluate changes in outcomes after implementation of a benzodiazepine-sparing AWS inpatient order set that included adjunctive therapies (eg, gabapentin, valproic acid, clonidine, and dexmedetomidine).

**Design, Setting, and Participants:**

This difference-in-differences quality improvement study was conducted among 22 899 AWS adult hospitalizations from October 1, 2014, to September 30, 2019, in the Kaiser Permanente Northern California integrated health care delivery system. Data were analyzed from September 2020 through November 2021.

**Exposures:**

Implementation of the benzodiazepine-sparing AWS order set on October 1, 2018.

**Main Outcomes and Measures:**

Adjusted rate ratios for medication use, inpatient mortality, length of stay, intensive care unit admission, and nonelective readmission within 30 days were calculated comparing postimplementation and preimplementation periods among hospitals with and without order set use.

**Results:**

Among 904 540 hospitalizations in the integrated health care delivery system during the study period, AWS was present in 22 899 hospitalizations (2.5%), occurring among 16 323 unique patients (mean [SD] age, 57.1 [14.8] years; 15 764 [68.8%] men). Of these hospitalizations, 12 889 (56.3%) used an order set for alcohol withdrawal. Among hospitalizations with order set use, any benzodiazepine use decreased after implementation from 6431 hospitalizations (78.1%) to 2823 hospitalizations (60.7%) (*P* < .001), with concomitant decreases in the mean (SD) total dosage of lorazepam before vs after implementation (19.7 [38.3] mg vs 6.0 [9.1] mg; *P* < .001). There were also significant changes from before to after implementation in the use of adjunctive medications, including gabapentin (2413 hospitalizations [29.3%] vs 2814 hospitalizations [60.5%]; *P* < .001), clonidine (1476 hospitalizations [17.9%] vs 2208 hospitalizations [47.5%]; *P* < .001), thiamine (6298 hospitalizations [76.5%] vs 4047 hospitalizations [87.0%]; *P* < .001), valproic acid (109 hospitalizations [1.3%] vs 256 hospitalizations [5.5%]; *P* < .001), and phenobarbital (412 hospitalizations [5.0%] vs 292 hospitalizations [6.3%]; *P* = .003). Compared with AWS hospitalizations without order set use, use of the benzodiazepine-sparing order set was associated with decreases in intensive care unit use (adjusted rate ratio [ARR], 0.71; 95% CI, 0.56-0.89; *P* = .003) and hospital length of stay (ARR, 0.71; 95% CI, 0.58-0.86; *P* < .001).

**Conclusions and Relevance:**

This study found that implementation of a benzodiazepine-sparing AWS order set was associated with decreased use of benzodiazepines and favorable trends in outcomes. These findings suggest that further prospective research is needed to identify the most effective treatments regimens for patients hospitalized with alcohol withdrawal.

## Key Points

**Question:**

Was implementation of a benzodiazepine-sparing order set associated with changes in medication use and outcomes for patients hospitalized with alcohol withdrawal syndrome?

**Findings:**

In this quality improvement study of 22 899 hospitalizations among 16 323 adults diagnosed with alcohol withdrawal syndrome, order set implementation was associated with relative decreases in benzodiazepine exposure, intensive care unit use, and length of stay.

**Meaning:**

The findings of this study suggest that quality improvement initiatives to decrease benzodiazepine use among patients with alcohol withdrawal syndrome may be associated with decreased use of benzodiazepines and positive patient health outcomes.

## Teaser

This quality improvement study investigates the association of a benzodiazepine-sparing order set at an integrated health care system with medication use and health care outcomes among patients with alcohol withdrawal syndrome.

## Introduction

Alcohol use disorders account for more than 400 000 hospitalizations each year with a total estimated cost of $3.5 billion in the US.^1^ Among inpatients with alcohol use disorders, alcohol withdrawal syndrome (AWS) occurs with an incidence between 2% and 7%.^2,3^ Up to 20% of individuals with AWS can develop severe complications, including autonomic instability, seizures, hallucinations, and delirium tremens, with mortality rates between 3% and 15%.^2,4,5^ While benzodiazepines (BZDs) are the mainstay of treatment,^5,6,7,8^ with symptom-triggered treatment recommended to decrease overall BZD exposure,^8,9,10,11,12^ it is unclear what protocols most hospitals use to treat AWS.

While BZDs are associated with effective reductions in AWS symptoms, they are also associated with adverse effects, including excessive sedation, falls, respiratory depression, aspiration, delirium, and even mortality.^10,13,14,15,16,17,18^ Alternate AWS treatments have been suggested, including anticonvulsants and α-2 adrenergic agonists.^19,20,21,22,23,24,25^ However, the evidence supporting these treatments is limited, with a handful of small prospective trials and retrospective studies suggesting benefit.^26,27,28,29,30,31,32^ Thus, significant uncertainty remains about the optimal approach to AWS.

Considering the risks associated with BZDs and the paucity of existing evidence for improving AWS care, the goal of this study was to evaluate the association of use of a revised protocol incorporating BZD-sparing (BZD-S) treatments with outcomes. Because order sets are widely used as tools to standardize prescribing choices within electronic health records (EHRs), we evaluated the association of a revised order set with medication practices and outcomes across 21 hospitals.^33^ This quality improvement effort was designed to promote continuous learning by systemically monitoring the safety, quality, and outcomes of care after protocol implementation.^34^ We hypothesized that the implementation of a BZD-S AWS inpatient order set would be associated with decreased BZD use and improved outcomes.

## Methods

In this quality improvement study, we conducted a retrospective evaluation of a quality improvement project for AWS in the Kaiser Permanente Northern California (KPNC) integrated health care delivery system, which serves 4.4 million members across 21 hospitals. This study was deemed to not be human participants research and exempt from institutional review board review and informed consent by the KPNC research determination officer. This study is reported following the Strengthening the Reporting of Observational Studies in Epidemiology (STROBE) reporting guideline.

### Development of a BZD-Sparing AWS Order Set

In 2015, KPNC physicians noted the potential for BZD-associated complications in 2 existing AWS order sets, one directed toward patients not in the intensive care unit (ICU) and another toward patients in the ICU (eTable 1 in the Supplement), which included only BZDs for prophylaxis or treatment. Physicians launched a quality -improvement project to incorporate literature-supported adjunctive treatments for AWS, including anticonvulsants (eg, carbamazepine, gabapentin, and valproic acid) and α-2 adrenergic agonists (eg, clonidine and dexmedetomidine) for prophylaxis and management of AWS.^4,21^ This work group comprised specialists from addiction medicine, critical care, hospital medicine, and consultation-liaison psychiatry who identified potential treatments for patients with AWS through evaluation of the scientific literature and review of local pilot projects. The work group developed a revised BZD-S order set for AWS through an iterative, consensus-based approach that streamlined AWS treatment for the ward and ICU while seeking to decrease overall BZD use. The order set was approved by the hospital medicine chiefs committee, a hospital order set content governance committee, and, lastly, the hospital medical executive committee for implementation across all hospital sites as a quality improvement initiative on October 1, 2018.

The revised BZD-S order set included cascading order set options based on 3 risk categories using a clinician’s determination of risk of complicated AWS based on the Prediction of Alcohol Withdrawal Severity Scale (PAWSS)^4^ or clinical severity based on the Clinical Institute Withdrawal Assessment for Alcohol, revised (CIWA-Ar)^9^ scale. PAWSS scores risk factors from patient history and clinical evidence (possible score range, 0-10). CIWA-Ar measures clinical severity based on 10 alcohol withdrawal symptoms (possible score range, 0-67). The categories represented clinical pathways, including observation, prevention or active withdrawal, and severe or complex withdrawal in the ICU (Table 1).^4,9^ Patients with low risk for AWS (ie, PAWSS score < 4) and low severity (ie, CIWA-Ar score < 8) could be placed on the observation pathway and given supportive treatment with thiamine and multivitamins. Patients at high risk for AWS (ie, PAWSS score ≥ 4) or those who were experiencing active AWS (ie, CIWA-Ar score ≥ 8 and < 15) could be placed into the prevention or active withdrawal pathway. Treatment options in this pathway included anticonvulsants (ie, gabapentin or valproic acid) and an α-agonist (ie, clonidine) as primary treatment, with BZDs as needed for breakthrough AWS (ie, CIWA-Ar score ≥ 15). Finally, patients presenting with severe AWS (ie, CIWA-Ar score ≥ 15) or those not responsive to other pathways were treated based on the severe and complex withdrawal pathway in the ICU. This included an option for dexmedetomidine, which requires frequent monitoring of cardiovascular adverse effects. Each successive clinical pathway incorporated orders from the less severe pathways.

**Table 1.** Elements of BZD-Sparing Order Set for Alcohol Withdrawal Syndrome

Pathway	Treatment	Dosing
Observation (criteria: PAWSS < 4 and CIWA-Ar < 8): thiamine, folic acid, multivitamin, and maintenance fluid	Thiamine^a^	Day 1-3: 200 mg by mouth 2 times daily
		Day 4+: 100 mg by mouth daily
	Folic acid^b^	Day 1+: 1 mg by mouth daily
	Multivitamin^a^	Day 1+: 1 tablet by mouth daily
	Maintenance fluid	Day 1+: 20 mEq/L potassium chloride in 5% dextrose and 0.45% sodium chloride at 100 mL/h by IV route continuously
Prevention and active withdrawal (criteria: PAWSS ≥ 4 and CIWA-Ar ≥ 8): gabapentin or valproic acid, then clonidine, then lorazepam, with treatment from observation pathway	Gabapentin	Loading dose: 1200 mg by mouth
		Day 1-3: 800 mg by mouth 3 times daily
		Day 4-5: 600 mg by mouth 3 times daily
		Day 6-7: 300 mg by mouth 3 times daily
	Gabapentin (low dose)	Loading dose: 1200 mg by mouth
		Day 1-5: 300 mg by mouth 3 times daily
		Day 6-10: 300 mg by mouth 2 times daily
		Day 11-20: 600 mg by mouth at bedtime
	Valproic acid^c^	Day 1-5: 250 mg by mouth 2 times daily
		Day 1-10: 500 mg by mouth at bedtime
	Clonidine	Day 1: 0.2 mg by mouth every 8 h for 2 doses and three 0.1 mg/24 h transdermal patches (0.3 mg total)
		Day 3: Remove one 0.1 mg/24 h transdermal patch
		Day 6: Remove one 0.1 mg/24 h transdermal patch
		Day 7: Remove one 0.1 mg/24 h transdermal patch
		Day 8: 0.1 mg/24 h transdermal patch
		Day 10: Remove one 0.1 mg/24 h transdermal patch
	Lorazepam^c^	CIWA-Ar score 16-20: 1 mg by mouth every 4 h as needed
		CIWA-Ar score >21: 2 mg by mouth every 4 h as needed
Severe and complex withdrawal (criteria: admitted to ICU with CIWA-Ar≥15 or not responsive to BZDs): dexmedetomidine with prevention and active withdrawal and observation pathways	Dexmedetomidine	Day 1+: 0.4 µg/kg/h by IV route continuously.
		Titrate by 0.2 µg/kg/h every 10-15 min up to a maximum of 1.4 µg/kg/h to achieve CIWA-Ar score <12 or RASS score −1 to 1.

Abbreviations: CIWA-Ar, Clinical Institute Withdrawal Assessment for Alcohol, revised^9^; ICU, intensive care unit; IV, intravenous; PAWSS, Prediction of Alcohol Withdrawal Severity Scale^4^; RASS, Richmond Agitation-Sedation Scale.

^a^ May be administered by IV or nasogastric routes if patient is unable to tolerate oral dosing.

^b^ May be administered by nasogastric route if patient is unable to tolerate oral dosing.

^c^ May be administered by IV route if patient is unable to tolerate oral dosing.

When using the order set, physicians selected 1 of the 3 pathways using manual assessments and documentation of the PAWSS or CIWA-Ar scores; no default pathway existed in the order set. The pathways also included options for intravenous medication administration if patients were unable to tolerate oral medications. The BZD-S order set further contained mandatory orders for fall-risk precautions, aspiration pneumonia precautions, and CIWA-Ar assessments every 4 to 6 hours, with optional orders for maintenance fluids or psychiatry or chemical dependency consultation requests. Finally, the BZD-S order set provided embedded education for clinicians on the timing of alcohol withdrawal. However, the order set was not mandated for the treatment of AWS, giving clinicians discretion to use the order set when clinically relevant.

### Cohort Formation

To evaluate outcomes associated with implementation of the BZD-S order set, we identified nonobstetric hospitalizations of adult (ie, patients aged ≥18 years) patients between October 1, 2014, and September 30, 2019, with AWS based *International Classification of Diseases, Ninth Revision, Clinical Modification* and *International Statistical Classification of Diseases, Tenth Revision, Clinical Modification* inpatient diagnosis codes (ie, 291.x, 303.x, 305.0, 790.3, and F10.x).

### Preintervention and Postintervention Periods

The BZD-S order set was implemented on October 1, 2018, and fully replaced prior order sets. Therefore, we defined the preimplementation period as the 4 consecutive years from October 1, 2014, to September 30, 2018, and the postimplementation period as the 1 year from October 1, 2018, to September 30, 2019.

### Outcome Measures

The primary outcome was inpatient mortality. Secondary outcomes included hospital length of stay (LOS), ICU admission, and hospital readmission within 30 days of hospital discharge (using the Healthcare Effectiveness Data and Information Set definition).^35^ We also examined changes in the use of AWS-related medications (eg, BZDs, clonidine, dexmedetomidine, gabapentin, phenobarbital, thiamine, and valproic acid) over the study period.

### Covariates

Patient characteristic data were extracted from EHRs^36^ and included age, sex, inpatient vs observation admission, weekday vs weekend admission, and facility. We quantified AWS characteristics based on the number of prior AWS-related hospitalizations within the prior 6 months, the first CIWA-Ar score recorded (if available), and the presence of urine toxicology screening to assess for concurrent drug use. First CIWA-Ar values were grouped as low (<8), medium (≥8 and <15), or high (≥15). Finally, we assessed patient severity of illness using 2 variables: the Laboratory and Acute Physiology Score, version 2 (LAPS2; possible score range, 0-292), which incorporates vital signs and laboratory results, and the Comorbidity Point Score, version 2 (COPS2; possible score range, 0-318), which scores chronic comorbid disease burden using diagnosis codes in the prior year.^36^ Physician-specific information was not captured, and if patient data were not present (eg, for medications or diagnoses), we assumed that they were not used.

### Statistical Analysis

We described patient and encounter characteristics using mean (SD), median (IQR), and frequency (No. [%]). We compared unadjusted outcomes between preintervention and postintervention groups among hospitalizations using *t*, Kruskal-Wallis, or χ^2^ tests. To evaluate the comparative association of BZD-S order set use with outcomes, we used a difference-in-differences approach with generalized estimating equation models to estimate differences for each outcome, including an indicator of period (ie, preimpementation vs postimplementation), an indicator for order set use (ie, yes vs no), and an interaction term between period and order set indicators (eMethods in Supplement). The main effects (ie, estimating the relative association of the order set across time periods) were based on the interaction term. For binary values, we used a Poisson distribution with a log-link function, and for continuous values, we used a γ distribution with a log-link function, with the results reported as rate ratios (RRs). To account for hospital variation and patients with multiple encounters, we treated hospitals as fixed effects, with an exchangeable within-patient correlation structure. We adjusted for age, sex, comorbidity (using COPS2) and acuity (using LAPS2) scores, prior AWS hospitalization in the past 6 months, observation vs inpatient admission, weekend admission, urine toxicology, and month of year and preintervention vs postintervention time trends.

We also conducted 3 sensitivity analyses among subcohorts, including only hospitalizations (1) in which patients received 1 or more AWS-relevant medications (ie, BZD, clonidine, dexmedetomidine, gabapentin, phenobarbital, or valproic acid), (2) in which patients received 1 or more BZDs, and (3) occurring during the 12-month period before or after implementation. Analyses used Python programming language version 3.7.3 (Python Software Foundation) or SAS statistical software version 14.5 (SAS Institute). We considered a 2-sided *P* value < .05 to be statistically significant. Data were analyzed from September 2020 through November 2021.

## Results

Among 904 540 hospitalizations in the integrated health care delivery system during the study period, we identified a total of 22 899 hospital visits with AWS (2.5%), occurring among 16 323 unique patients with AWS (mean [SD] age, 57.1 [14.8] years; 15 764 men [68.8%] men) (Table 2).^9,36^ Of this total, 12 889 hospitalizations (56.3%) included the use of an order set for alcohol withdrawal: 8237 of 14 538 preimplementation hospitalizations (56.7%) and 4652 of 8361 postimplementation hospitalizations (55.6%) (eFigure in the Supplement). There were 10 010 hospitalizations without order-set use (including 6301 preimplementation hospitalizations [43.3%] and 3709 postimplementation hospitalizations [44.4%]). Among hospitalizations with order set use, those occurring before BZD-S order set implementation vs those after implementation had younger patients (mean [SD] age, 54.1 [14.0] years vs 54.7 [14.7] years; *P* = .02) and decreased acute severity of illness (mean [SD] LAPS2 score, 66.2 [38.1] vs 73.8 [39.7]; *P* < .001). Additionally, among hospitalizations with use of an order set, those before order set implementation had decreased rates of urine toxicology testing (3269 hospitalizations [39.7%] vs 2129 hospitalizations [45.8%]; *P* < .001) and history of alcohol abuse diagnoses (6845 hospitalizations [83.1%] vs 4145 hospitalizations [89.1%]; *P* < .001). Decreased severity of illness and increased alcohol-related use were also present among AWS hospitalizations without order set use in the preimplementation period compared with postimplementation (Table 2).

**Table 2.** Baseline Characteristics of Hospitalizations for AWS

Characteristic	Hospitalizations, No. (%)						
	With use of order set (n = 12 889)			Without use of order set (n = 10 010)			Overall (N = 22 899)
	Before implementation (n = 8237)	After implementation (n = 4652)	*P* value	Before implementation (n = 6301)	After implementation (n = 3709)	*P* value	
Age, mean (SD), y	54.1 (14.0)	54.7 (14.7)	.02	60.2 (14.4)	61.7 (15.3)	<.001	57.1 (14.8)
Sex							
Men	5739 (69.7)	3244 (69.7)	.94	4306 (68.3)	2475 (66.7)	.10	15 764 (68.8)
Women	2498 (30.3)	1408 (30.3)		1995 (31.7)	1234 (33.3)		7135 (31.2)
Observation admission	813 (9.9)	327 (7.0)	<.001	399 (6.3)	94 (2.5)	<.001	1633 (7.1)
Weekend admission	2161 (26.2)	1239 (26.6)	.62	1001 (15.9)	795 (21.4)	<.001	5196 (22.7)
LAPS2, mean (SD)	66.2 (38.1)	73.8 (39.7)	<.001	48.6 (42.3)	69.4 (45.8)	<.001	63.4 (42.0)
COPS2, mean (SD)	39.7 (43.2)	40.4 (46.9)	.37	55.8 (53.7)	63.1 (62.0)	<.001	48.0 (51.2)
Urine toxicology checked	3269 (39.7)	2129 (45.8)	<.001	848 (13.5)	777 (20.9)	<.001	7023 (30.7)
Had ≥1 prior hospitalization for AWS within prior 6 mo	2611 (31.7)	1545 (33.2)	.08	1121 (17.8)	719 (19.4)	.047	5996 (26.2)
Had ≥1 CIWA-Ar value recorded	8167 (99.2)	4525 (97.3)	<.001	551 (8.7)	159 (4.3)	<.001	13 402 (58.5)
Elixhauser comorbidities^a^							
Alcohol abuse	6845 (83.1)	4145 (89.1)	<.001	6122 (97.2)	3654 (98.5)	<.001	20 766 (90.7)
Hypertension^b^	4853 (58.9)	2814 (60.5)	.13	4104 (65.1)	2555 (68.9)	<.001	14 326 (62.6)
Fluid and electrolyte disorder	4867 (59.1)	2942 (63.2)	<.001	2625 (41.7)	2086 (56.2)	<.001	12 520 (54.7)
Peripheral vascular disease	2956 (35.9)	2006 (43.1)	<.001	3223 (51.2)	2335 (63.0)	<.001	10 520 (45.9)
Liver disease	3473 (42.2)	2229 (47.9)	<.001	2080 (33.0)	1515 (40.8)	<.001	9297 (40.6)

Abbreviations: AWS, alcohol withdrawal syndrome; CIWA-Ar, Clinical Institute Withdrawal Assessment for Alcohol, revised^9^; COPS2, Comorbidity Point Score, version 2^36^; LAPS2, Laboratory Acute Physiology Score, version 2.^36^

^a^ Includes the 5 most common comorbidities.

^b^ Includes complicated and uncomplicated hypertension.

**Figure 1.**
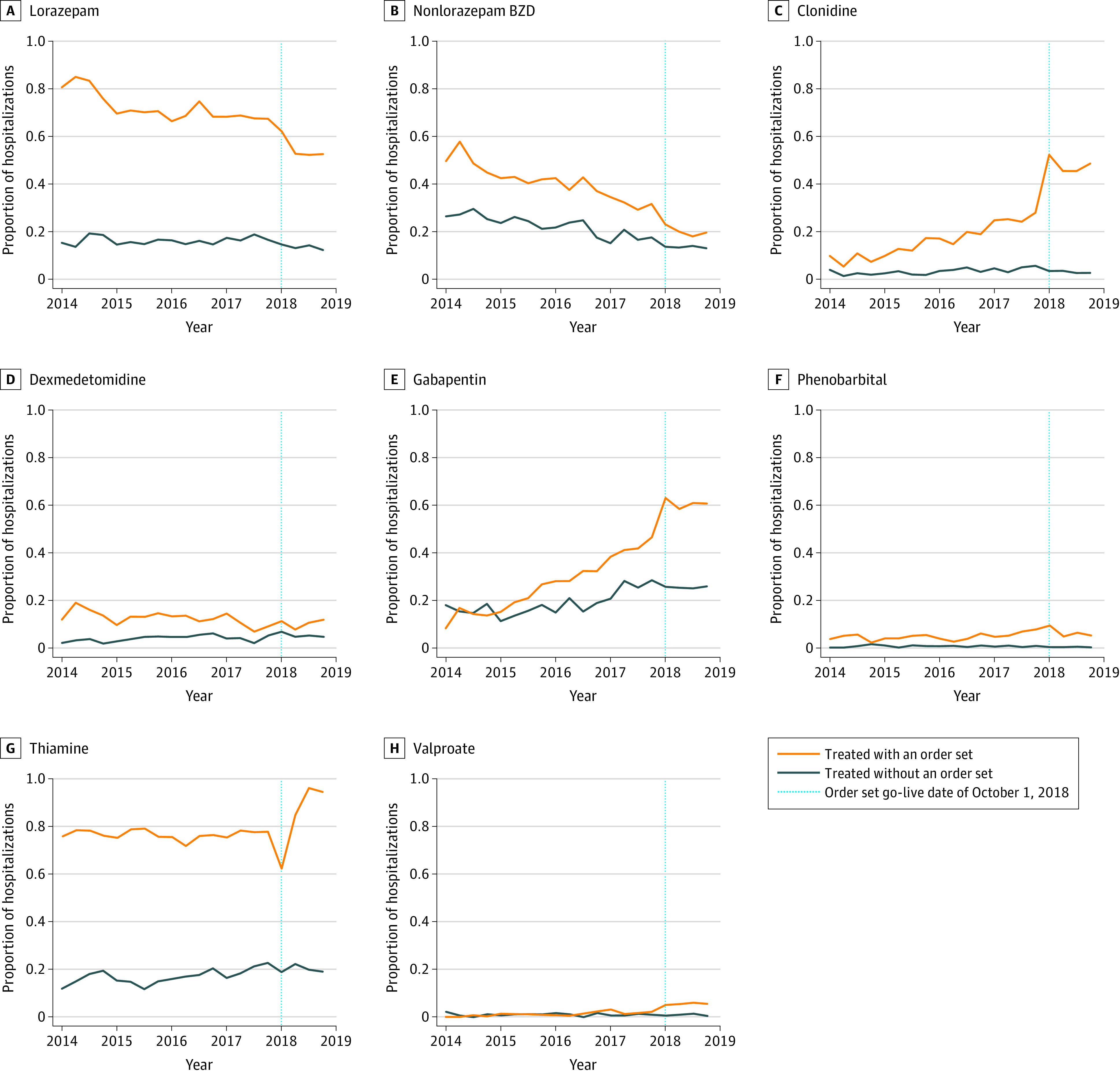
Hospitalizations With Alcohol Withdrawal Syndrome Medications Administered The proportion of hospitalizations with medications administered are presented over quarterly intervals before and after implementation of a benzodiazepine (BZD)–sparing alcohol withdrawal order set. Orange lines indicate hospitalizations with an order set; blue lines, hospitalizations without an order set; dotted lines, order set go-live date of October 1, 2018.

BZD-S order set use was associated with significant changes in medication treatment patterns for AWS when compared with hospitalizations using prior order sets (Figure 1). For example, among hospitalizations with order set use, administration of any BZD occurred among 6431 hospitalizations (78.1%) before implementation compared with 2823 hospitalizations (60.7%) after implementation (*P* < .001), with use decreasing prior to implementation followed by a greater decrease after implementation. Among hospitalizations with order set use with patients receiving lorazepam, there was also a significant decrease in the mean (SD) total dosage of lorazepam given from before to after implementation (19.7 [38.3] mg vs 6.0 [9.1] mg; *P* < .001). There were significant increases from before to after implementation in use of gabapentin (2413 hospitalizations [29.3%] vs 2814 hospitalizations [60.5%]; *P* < .001), clonidine (1476 hospitalizations [17.9%] vs 2208 hospitalizations [47.5%]; *P* < .001), thiamine (6298 hospitalizations [76.5%] vs 4047 hospitalizations [87.0%]; *P* < .001), valproic acid (109 hospitalizations [1.3%] vs 256 hospitalizations [5.5%]; *P* < .001), and phenobarbital administration (412 hospitalizations [5.0%] vs 292 hospitalizations [6.3%]; *P* = .003). Changes in medication administration among hospitalizations without order set use were also present but were smaller. For example, any lorazepam use decreased from 1017 preimplementation hospitalizations (16.1%) to 495 postimplementation hospitalizations (13.4%) (*P* < .001), while nonlorazepam use decreased from 1420 preimplementation hospitalizations (22.5%) to 540 postimplementation hospitalizations [14.6%] (*P* < .001).

**Figure 2.**
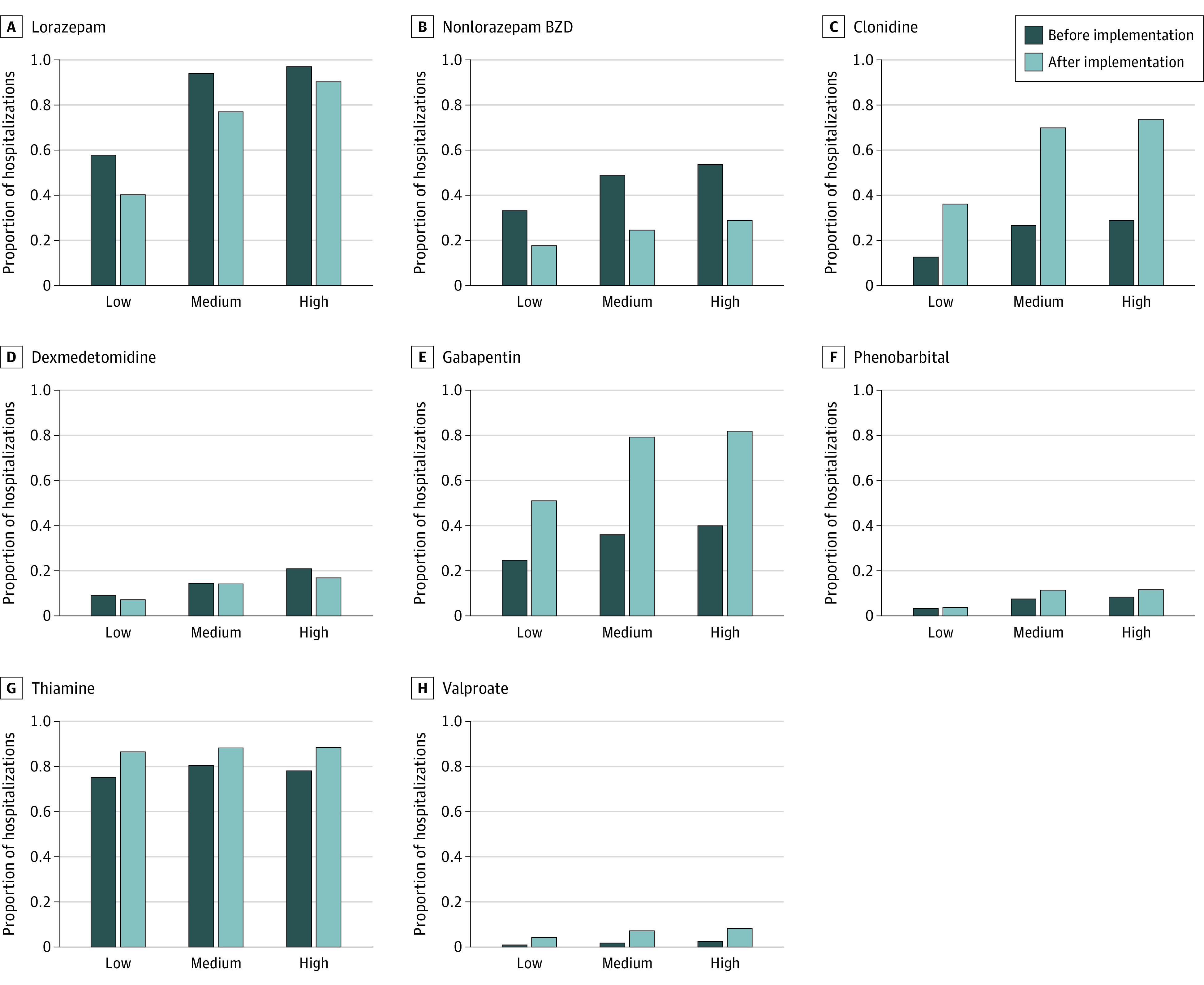
Hospitalizations With Alcohol Withdrawal Medications Administered and Order Set Used The proportion of hospitalizations with medications administered and order sets used before and after implementation are presented. Outcomes are stratified by first Clinical Withdrawal Assessment for Alcohol, revised values, which were grouped as low (ie, <8), medium (ie, ≥8 and <15), or high (ie, ≥15). BZD indicates benzodiazepine.

Most hospitalizations with order set use had at least 1 CIWA-Ar recording (8167 preimplementation hospitalizations [99.2%] and 4525 postimplementation hospitalizations [97.3%]). Among 13 402 hospitalizations with and without an order set with a CIWA-Ar value, there were 8735 hospitalizations (65.2%) with low, 2577 hospitalizations (19.2%) with medium, and 2090 hospitalizations (15.6%) with high values. Medication changes were consistent across severity groups, although the magnitude of change varied (Figure 2). For example, among hospitalizations with low first CIWA-Ar recordings, lorazepam administration decreased from 3245 of 5615 preimplementation hospitalizations (57.8%) to 1254 of 3120 postimplementation hospitalizations (40.2%) (*P* < .001), while nonlorazepam BZD administration decreased from 1836 of 5615 preimplementation hospitalizations (32.7%) to 534 of 3120 postimplementation hospitalizations (17.1%) (*P* < .001). Among hospitalizations with high first CIWA-Ar values, lorazepam use decreased from 1305 of 1345 prehospitalizations (97.0%) to 673 of 745 postimplementation hospitalizations (90.3%) (*P* < .001), while nonlorazepam BZD use decreased from 714 of 1345 preimplementation hospitalizations (53.1%) to 209 of 745 postimplementation hospitalizations (28.0%) (*P* < .001). Gabapentin administration increased from 1393 of 5615 preimplementation hospitalizations (24.8%) to 1594 of 3120 postimplementation hospitalizations (51.1%) among those with low CIWA-Ar values (*P* < .001), while it increased from 538 of 1345 preimplementation hospitalizations (40.0%) to 610 of 745 postimplementation hospitalizations to (81.9%) among those with high values (*P* < .001).

Unadjusted mortality increased from the preimplementation to the postimplementation period among AWS hospitalizations using order sets (107 hospitalizations [1.3%] vs 93 hospitalizations [2.0%]; *P* = .002) and those not using order sets (126 hospitalizations [2.0%] vs 166 hospitalizations [4.5%]; *P* < .001) (Table 3). Among hospitalizations using the order set, median (IQR) hospital LOS did not change from preimplementation to postimplementation (2.9 [1.8-5.0] days vs 2.9 [1.8-4.9] days; *P* = .44), while ICU use (2310 hospitalizations [28.0%] vs 1229 hospitalizations [26.4%]; *P* = .047) and readmissions (1433 hospitalizations [17.6%] vs 718 hospitalizations [15.7%]; *P* = .007) decreased. Among AWS hospitalizations without order set use, median (IQR) LOS (2.8 [1.4-5] days vs 3.2 [1.9-5.9] days; *P* < .001), ICU rates (1284 hospitalizations [20.4] vs 841 hospitalizations [22.7]; *P* = .007), and readmission rates (1191 hospitalizations [19.3]vs 766 hospitalizations [21.6]; *P* = .006) increased from preimplementation to postimplementation. Outcomes varied by first CIWA-Ar category (eTable 2 in the Supplement); for example, mortality was lowest in the high CIWA-Ar group, while ICU use was highest in the same group.

**Table 3.** Rate Ratios Preimplementation and Postimplementation by Order Set Use

Characteristic	Hospitalizations, No. (%)^a^						Difference in differences			
	With use of order set			Without use of order set			Unadjusted RR (95% CI)	*P* value	Adjusted RR (95% CI)^b^	*P* value
	Before implementation (n = 8237)	After implementation (n = 4652)	*P* value	Before implementation (n = 6301)	After implementation (n = 3709)	*P* value				
Inpatient mortality	107 (1.3)	93 (2.0)	.002	126 (2.0)	166 (4.5)	<.001	0.69 (0.48-0.98)	.04	0.86 (0.37-1.97)	.72
Hospital LOS, median (IQR), d	2.9 (1.8-5.0)	2.9 (1.8-4.8)	.44	2.8 (1.4-5.0)	3.2 (1.9-5.9)	<.001	0.84 (0.78-0.91)	<.001	0.71 (0.58-0.86)	<.001
ICU admission	2310 (28.0)	1229 (26.4)	.047	1284 (20.4)	841 (22.7)	.007	0.85 (0.78-0.94)	.001	0.71 (0.56-0.89)	.003
Readmission	1433 (17.6)	718 (15.7)	.007	1191 (19.3)	766 (21.6)	.006	0.93 (0.79-1.08)	.34	0.80 (0.59-1.08)	.14

Abbreviations: COPS2, Comorbidity Point Score, version 2^36^; ICU, intensive care unit; LAPS2, Laboratory Acute Physiology Score, version 2^36^; LOS, length of stay; RR, rate ratio.

^a^ Preimplementation and postimplementation period data are presented as unadjusted number (percentage) of encounters for binary outcomes and as mean (SD) for hospital LOS.

^b^ Adjusted for age, sex, comorbidity (COPS2) and acuity (LAPS2) scores, prior alcohol withdrawal syndrome hospitalization in the prior 6 months, observation vs inpatient admission, weekend admission, urine toxicology, and month of year and preintervention vs postimplementation time trends.

Using a difference-in-differences approach, we found that use of the BZD-S order set was not associated with a greater decrease in hospital mortality (adjusted rate ratio [ARR], 0.86; 95% CI, 0.37 to 1.97; *P* = .72) (Table 3) compared with hospitalizations in which the order set was not used. Nor was it associated with a decrease in readmission (ARR, 0.80; 95% CI, 0.59 to 1.08; *P* = .14). However, BZD-S order set use was associated with a relative decrease in ICU use (ARR, 0.71; 95% CI, 0.56 to 0.89; *P* = .003) and LOS (ARR, 0.71; 95% CI, 0.58 to 0.86; *P* < .001). Results were directionally similar in sensitivity analyses, although differences were not statistically significant across outcomes (eTables 3-6 in the Supplement).

## Discussion

As a learning hospital system that seeks to systematically evaluate the association of inpatient intervention with patient outcomes,^37^ we sought in this quality improvement study to evaluate outcomes after implementation of a quality improvement initiative using a BZD-sparing order set for AWS designed to reduce BZD exposure across 21 community-based hospitals. Compared with AWS hospitalizations with no order set implementation, BZD-S order set implementation was associated with a decrease in BZD administration and an increase in use of clonidine, gabapentin, phenobarbital, thiamine, and valproic acid. There were also favorable trends in all outcomes, with a statistically significant decrease in ICU use and LOS in our primary analysis. Taken together, these findings represent new evidence to inform the treatment of AWS, a condition in which the standard of care has a limited evidence base and has changed little over several decades.^38^

While many individuals have postulated that BZD reduction may be associated with improved outcomes in AWS, prior studies included smaller cohorts and focused on symptom-based outcomes with adjunctive AWS agents rather than on other hospital outcomes.^39,40^ In addition, most studies have examined the isolated outcomes associated with a single therapy rather than a combination of treatments.^19,20,22,23,24,25,26,27,29,31,32,40,41,42^ Nonetheless, the use of protocolized approaches has been recommended and put into practice primarily in academic medical centers.^21^ Large-scale outcomes studies including multiple therapies in community-based practice settings remain poorly described.^43^

Based on a multidisciplinary collaboration among diverse clinical specialties, the KPNC BZD-S order set contained a bundle of features, including educational information on AWS timing, a severity scale, thiamine dosing, several adjunctive agents, and reduced BZD dosing scales. The use of AWS adjunctive agents increased with a concurrent decrease in use and dose of BZDs. However, it should be noted that decreased BZD use was present before BZD-S order set implementation in 2018 and were also seen in AWS hospitalizations without order set use. These preexisting decreases may be associated with changing inpatient practice designed to limit BZD negative sequelae among inpatients and may not be associated with order set implementation alone. In addition, BZDs were still administered in most AWS episodes, particularly among patients with medium and high CIWA-Ar scores, suggesting that these drugs continue to play an important role in AWS treatment.

We observed favorable trends in our outcome measures and statistically significant relative reductions in ICU use and LOS. Interestingly, in all AWS hospitalizations, mortality increased after implementation, which may be associated with a changing case mix of patients hospitalized with AWS. The increased acuity is corroborated by substantially increased acute severity of illness metrics after 2018. While this mortality increase complicates the interpretation of our findings, this study’s difference-in-differences approach facilitates an assessment of the BZD-S order set against a changing background of outcomes. Additional prospective interventional studies are needed to evaluate the association of BZD-S protocols with patient outcomes and to investigate the relative association of individual elements within larger care bundles.

Our study has several strengths. First, we analyzed outcomes from a large, multicenter cohort of community-based adult hospitalizations in an integrated health care system with excellent longitudinal data capture. To our knowledge, this represents the largest study to date on the association of a BZD-S intervention with outcomes in the treatment of AWS. Second, we looked at outcomes from a multipart order set designed to improve care across several domains. Third, we evaluated important clinical outcomes, including mortality, ICU admissions, and LOS, rather than only symptoms or BZD use, which may help identify opportunities in the index and subsequent care for a condition that was present in 1 of 40 hospitalizations in our system.

### Limitations

Our study also has several limitations. First, our findings are subject to discrepancies from inaccurate documentation and use of order sets in a heterogeneous population. However, after identifying hospital encounters through diagnosis codes and AWS order sets, we confirmed a high prevalence of alcohol abuse diagnoses or a CIWA-Ar scores. Second, order set use was not mandated, limiting our ability to ensure that groups defined based on preimplementation vs postimplementation period or order set use were similar. Third, the mechanism for observed differences in outcomes is unknown and could be confounded by concurrent interventions to improve hospital care. Fourth, our intervention date was the date of regional implementation of the BZD-S order set, which did not account for potential outcomes associated with pilot sites or practice exhibited by individual clinicians during the preintervention period. Fifth, few rigorous and contemporary studies exist that identify the most efficacious treatments for AWS; thus, our multidisciplinary clinician team designed the protocol based on expert guidance. Other medications not included in the order set may be effective for treating AWS and should be evaluated in prospective studies or in health system quality-improvement programs. Sixth, our data did not include readmissions that started and ended outside KPNC hospitals, which could impact our findings. Seventh, we did not have information on the ordering physicians and could not account for a physician effect in our analyses. Eighth, we evaluated a one-year period after implementation; longer follow-up periods are needed.

## Conclusions

Regional implementation of an order set to treat AWS with BZD-S alternatives across 21 hospitals in an integrated health care system was associated with a greater decrease in the use of BZDs and increase in the use of adjunctive therapies compared with hospitalizations without order set use. While our evaluation suggests that the postimplementation order set was associated with a decrease in ICU admission and hospital LOS, future prospective studies are needed to confirm these findings.

## Notes

Supplement. **eTable 1.** Elements of Preimplementation Non–Intensive Care Unit Order Set for Alcohol Withdrawal Syndrome **eFigure 1.** Proportion of Potential Patients With Alcohol Withdrawal and Alcohol Withdrawal Order Set Use **eTable 2.** Outcomes by First Clinical Institute Withdrawal Assessment for Alcohol, Revised Score Value Category **eTable 3.** Difference-in-Differences Adjusted Outcomes in Sensitivity Analyses **eTable 4.** Baseline Characteristics of Hospitalizations for Alcohol Withdrawal Syndrome With Use of Alcohol Withdrawal-Relevant Medication **eTable 5.** Baseline Characteristics of Hospitalizations for Alcohol Withdrawal Syndrome With Use ≥1 Benzodiazepine **eTable 6.** Baseline Characteristics of Hospitalizations for Alcohol Withdrawal Syndrome 12 mo Before and After Implementation **eMethods.** SAS Code for Difference-in-Differences Analysis
