# Supplementary material for: Outcomes After Implementation of a Benzodiazepine-Sparing Alcohol Withdrawal Order Set in an Integrated Health Care System
Source: JAMA Netw Open. 2022 Feb 22;5(2):e220158. doi: 10.1001/jamanetworkopen.2022.0158 (PMC8864512; doi:10.1001/jamanetworkopen.2022.0158)
Supplement: Supplement. — eTable 1. Elements of Preimplementation Non–Intensive Care Unit Order Set for Alcohol Withdrawal Syndrome eFigure 1. Proportion of Potential Patients With Alcohol Withdrawal and Alcohol Withdrawal Order Set Use eTable 2. Outcomes by First Clinical Institute Withdrawal Assessment for Alcohol, Revised Score Value Category eTable 3. Difference-in-Differences Adjusted Outcomes in Sensitivity Analyses eTable 4. Baseline Characteristics of Hospitalizations for Alcohol Withdrawal Syndrome With Use of Alcohol Withdrawal-Relevant Medication eTable 5. Baseline Characteristics of Hospitalizations for Alcohol Withdrawal Syndrome With Use ≥1 Benzodiazepine eTable 6. Baseline Characteristics of Hospitalizations for Alcohol Withdrawal Syndrome 12 mo Before and After Implementation eMethods. SAS Code for Difference-in-Differences Analysis [file jamanetwopen-e220158-s001.pdf]

Supplemental Online Content

Smith JT, Sage M, Szeto H, et al. Outcomes after implementation of a benzodiazepine-sparing alcohol withdrawal order set in an integrated health care system. *JAMA Netw Open*. 2022;5(2);e220158. doi:10.1001/jamanetworkopen.2022.0158

eTable 1. Elements of Preimplementation Non–Intensive Care Unit Order Set for Alcohol Withdrawal Syndrome

eFigure 1. Proportion of Potential Patients With Alcohol Withdrawal and Alcohol Withdrawal Order Set Use

eTable 2. Outcomes by First Clinical Institute Withdrawal Assessment for Alcohol, Revised Score Value Category

eTable 3. Difference-in-Differences Adjusted Outcomes in Sensitivity Analyses

eTable 4. Baseline Characteristics of Hospitalizations for Alcohol Withdrawal Syndrome With Use of Alcohol Withdrawal-Relevant Medication

eTable 5. Baseline Characteristics of Hospitalizations for Alcohol Withdrawal Syndrome With Use ≥1 Benzodiazepine

eTable 6. Baseline Characteristics of Hospitalizations for Alcohol Withdrawal Syndrome 12 mo Before and After Implementation

eMethods. SAS Code for Difference-in-Differences Analysis

This supplemental material has been provided by the authors to give readers additional information about their work.

© 2022 Smith JT et al. *JAMA Network Open.*

eTable 1a. Elements of Preimplementation Non–Intensive Care Unit Order Set for Alcohol Withdrawal Syndrome

Pathway	Treatment	Dosing
**Observation (Criteria: CIWA-Ar <8)**<br>Measure CIWA-Ar every 6 hours with no medication orders		
**Withdrawal Prophylaxis (Criteria: CIWA-Ar <8)**<br>Measure CIWA-Ar every 6 hours with diazepam or lorazepam		
	Diazepam (mild risk)	Day 1: 10 mg by mouth every 8 hours<br>Day 2: 10 mg by mouth every 12 hours<br>Day 3: 10 mg by mouth every daily
	Lorazepam (mild risk)[a]	Day 1: 1 mg by mouth every 8 hours<br>Day 2: 1 mg by mouth every 12 hours<br>Day 3: 1 mg by mouth every daily
	Diazepam (moderate risk)	Day 1: 10 mg by mouth every 6 hours<br>Day 2: 10 mg by mouth every 8 hours<br>Day 3: 10 mg by mouth every 12 hours
	Lorazepam (moderate risk)[a]	Day 1: 1 mg by mouth every 6 hours<br>Day 2: 1 mg by mouth every 8 hours<br>Day 3: 1 mg by mouth every 12 hours
	Diazepam (severe risk)	Day 1 to 2: 10 mg by mouth every 6 hours<br>Day 3: 10 mg by mouth every 8 hours<br>Day 4: 10 mg by mouth every 12 hours
	Lorazepam (severe risk)[a]	Day 1: 2 mg by mouth every 6 hours<br>Day 2: 2 mg by mouth every 8 hours<br>Day 3: 2 mg by mouth every 12 hours
**Active Withdrawal Treatment (Criteria: CIWA-Ar ≥8)**<br>Measure CIWA-Ar every 4 hours with diazepam or lorazepam		
	Diazepam	CIWA-Ar score 8-13: 10 mg by mouth every hour as needed<br>CIWA-Ar score 14-20: 15 mg by mouth every hour as needed<br>CIWA-Ar score ≥21: 20 mg by mouth every hour as needed
	Lorazepam[a]	CIWA-Ar score 8-13: 1 mg by mouth every hour as needed<br>CIWA-Ar score 14-20: 2 mg by mouth every hour as needed<br>CIWA-Ar score ≥21: 4 mg by mouth every hour as needed
**IV Fluids and Other Medications**<br>Thiamine, folic acid, multivitamin, and maintenance fluid		
	Thiamine	Day 1 to 5: 100 mg by mouth 2 times daily
	Folic Acid	Day 1+: 1 mg by mouth daily
	Multivitamin	Day 1+: 1 tablet by mouth daily
	Maintenance fluid	Day 1+: 20 mEq/L potassium chloride in 5% dextrose and 0.45% sodium chloride at 100 mL/hr by IV route continuously

[a]May be administered by IV route if patient is unable to tolerate oral dosing.

Abbreviations: CIWA-Ar, Clinical Institute Withdrawal Assessment for Alcohol, revised.[8]

© 2022 Smith JT et al. *JAMA Network Open.*

eTable 1b. Elements of Preimplementation Non–Intensive Care Unit Order Set for Alcohol Withdrawal Syndrome

**ICU Acute Alcohol Withdrawal**<br>Discontinue any other sedatives, hypnotics, central nervous system depressants, or tranquilizers<br><br>Measure CIWA-Ar every 15 minutes after every lorazepam dose, every 2 hours while awake, and 4 hours while asleep
**Loading**<br>1. Start lorazepam using *Dose Scale A* based on CIWA-Ar<br>2. Reassess CIWA-Ar score 15 minutes after administering the 1st dose and if needed give 2nd dose per *Dose Scale A*<br>3. Reassess CIWA-Ar score 30 minutes after administering the 1st dose<br>4. If CIWA-Ar score is ≤15, continue to use *Dose Scale A*<br>5. If CIWA-Ar score if >15, move to *Dose Scale B* and administer lorazepam dose per protocol
**Monitoring**<br>Measure CIWA-Ar every 15 minutes after every lorazepam dose, every 2 hours while awake, and 4 hours while asleep
**Escalation**<br>1. If using *Dose Scale A* and 2 doses of lorazepam within 30 minutes fail to improve a CIWA-AR score of greater than 15, move to *Dose Scale B* and give Lorazepam dose per protocol<br>2. If using *Dose Scale B* and 2 doses of lorazepam within 30 minutes fail to improve a CIWA-Ar score of greater than 20, notify physician

CIWA-Ar Score	Dose Scale A	Dose Scale B
>20	4 mg IV	6 mg IV
16-20	3 mg IV	5 mg IV
11-15	2 mg IV	4 mg IV
6-10	1 mg IV	3 mg IV
0-5	0 mg IV	0 mg IV

[a]May be administered by IV route if patient is unable to tolerate oral dosing.
Abbreviations: CIWA-Ar, Clinical Institute Withdrawal Assessment for Alcohol, revised;[8] ICU, intensive care unit.

© 2022 Smith JT et al. *JAMA Network Open.*

eFigure 1. Proportion of Potential Patients With Alcohol Withdrawal and Alcohol Withdrawal Order Set Use

The blue circles represent quarterly proportions while the red line displays a smooth fitted line. The dotted black line represents the implementation of the novel benzodiazepine-sparing orderset.

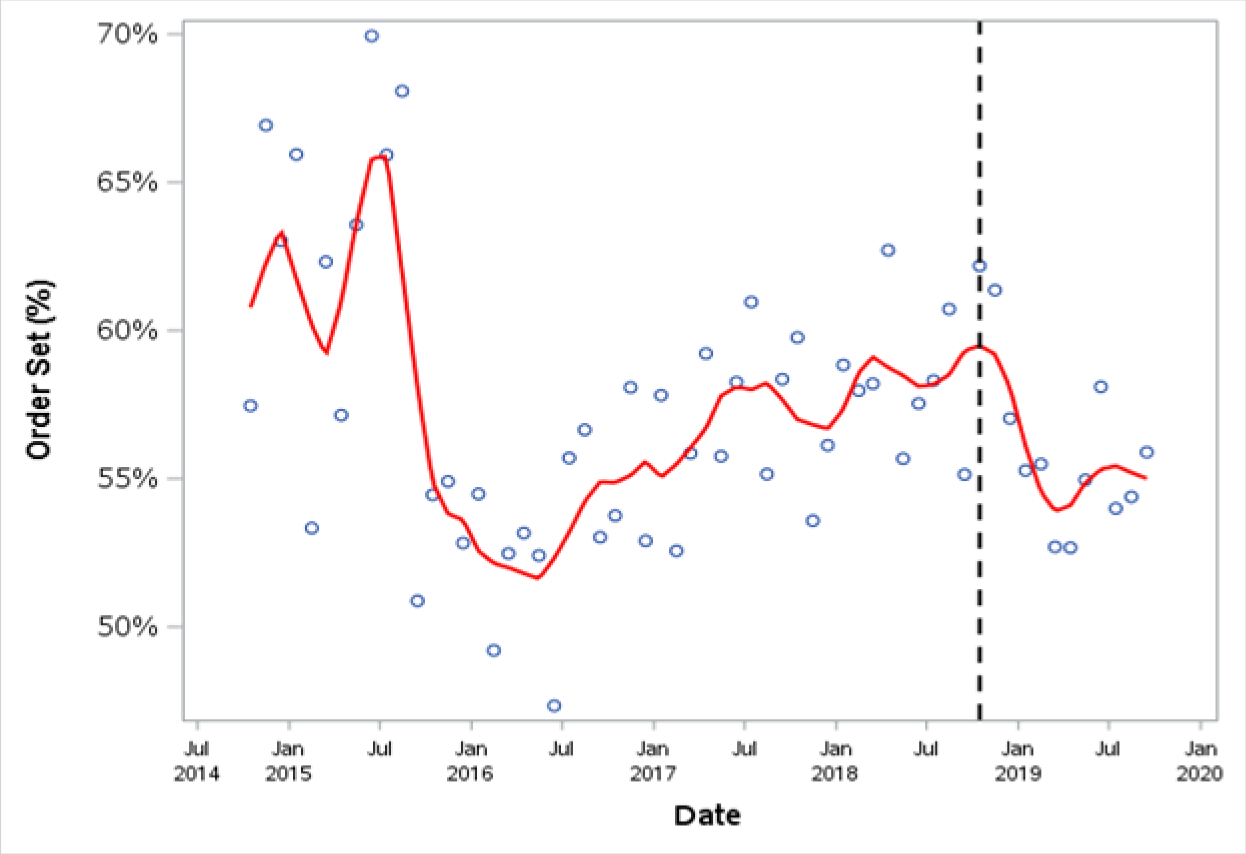

© 2022 Smith JT et al. *JAMA Network Open.*

eTable 2. Outcomes by First Clinical Institute Withdrawal Assessment for Alcohol, Revised Score Value Category

	First CIWA-Ar value			
	**Low (<8)**	**Medium (8-14)**	**High (≥15)**	***P* value**
Inpatient mortality	145 (1.7)	24 (0.9)	16 (0.8)	.001
Length of stay, days	4.7 ± 7.6	4.5 ± 6.1	4.6 ± 6.7	.32
ICU admission	2,213 (25.3)	692 (26.9)	711 (34.0)	<.001
Readmission	1,463 (17.0)	432 (16.9)	354 (17.1)	.99

Abbreviations: CIWA-Ar, Clinical Institute Withdrawal Assessment for Alcohol, revised;[8] ICU, intensive care unit.

© 2022 Smith JT et al. *JAMA Network Open.*

eTable 3. Difference-in-Differences Adjusted Outcomes in Sensitivity Analyses[a]

Including hospitalizations: (1) with at least on alcohol withdrawal-relevant medication administration[b]; (2) with at least one benzodiazepine administered[c]; and (2) within the 12-month periods before and after implementation.

	Difference-in-differences change		
**Outcome**	**At least one AWS-relevant medication given**	**At least one benzodiazepine given**	**12 month pre- and post- periods**
Inpatient mortality	0.70 (0.28-1.75); *P*=.45	0.70 (0.26-1.88); *P*=.48	0.68 (0.52-0.89); *P*=.005
Length of stay, days[d]	0.65 (0.50-0.85); *P*=.001	0.56 (0.41-0.76); *P*<.001	0.96 (0.93-0.99); *P*=.02
ICU admission	0.65 (0.50 – 0.84); *P*=.001	0.56 (0.42-0.75); *P*<.001	1.00 (0.91 – 1.10); *P*=.97
Readmission	0.70 (0.48-1.04); *P*=.07	0.61 (0.38-0.98); *P*=.04	0.88 (0.79-0.98); *P*=.02[e]

[a]Adjusted models include age, gender, COPS2, LAPS2, prior alcohol withdrawal hospitalization, maximum CIWA-Ar value, CIWA-Ar value recordings, observation status, month of year, weekend admission, urine toxicology assessed, and facility.
[b]Includes any benzodiazepine, clonidine, dexmedetomidine, gabapentin, phenobarbital, and/or valproic acid.
[c]Includes alprazolam, chlordiazepoxide, diazepam, lorazepam, midazolam, oxazepam, and/or temazepam.
[d]Assumed a gamma distribution and log-link in the GEE model.
[e]Parallel slopes in pre-period assumption not met.
Abbreviations: AWS, alcohol withdrawal; CIWA-Ar, Clinical Institute Withdrawal Assessment for Alcohol, revised;[8] CI, confidence interval; COPS2, Comorbidity Point Score, version 2;[29] ICU, intensive care unit; LAPS2, Laboratory Acute Physiology Score, version 2;[29]."

© 2022 Smith JT et al. *JAMA Network Open.*

eTable 4. Baseline Characteristics of Hospitalizations for Alcohol Withdrawal Syndrome With Use of Alcohol Withdrawal-Relevant Medication[a]

	With use of an orderset		Without use of an orderset	
**Characteristic**	**Pre-implementation (n = 6,835)**	**Post-implementation (n = 3,696)**	**Pre-implementation (n = 2,987)**	**Post-implementation (n = 1,680)**
Age, mean (SD), years	53.1 (13.5)	53.6 (14.2)	58.8 (13.9)	60.0 (14.6)
Male, (%)	4,753 (69.5)	2,556 (69.2)	1,942 (65.0)	1,075 (64.0)
Observation admission, (%)	714 (10.4)	298 (8.1)	186 (6.2)	41 (2.4)
Weekend admission, (%)	1,791 (26.2)	970 (26.2)	497 (16.6)	320 (19.0)
LAPS2, mean (SD)	68.2 (37.5)	74.6 (39.7)	50.6 (43.7)	70.2 (48.2)
COPS2, mean (SD)	40.2 (43.3)	41.4 (47.5)	58.7 (55.4)	65.9 (64.6)
Urine toxicology checked (%)	2,934 (42.9)	1,802 (48.8)	496 (16.6)	409 (24.3)
Had ≥1 prior hospitalization for AWS within prior 6 months, (%)	2,345 (34.3)	1,333 (36.1)	632 (21.2)	377 (22.4)
Had ≥1 CIWA-Ar value recorded, (%)	6,798 (99.5)	3,610 (97.7)	439 (14.7)	100 (6.0)
**Elixhauser comorbidities[b]**				
Alcohol abuse, (%)	5,495 (80.4)	3,204 (86.7)	2,864 (95.9)	1,655 (98.5)
Hypertension[c], (%)	3,984 (58.3)	2,212 (59.8)	1,945 (65.1)	1,157 (68.9)
Fluid and electrolyte disorders, (%)	4,212 (61.6)	2,406 (65.1)	1,355 (45.4)	1,001 (59.6)
Peripheral vascular disease, (%)	2,347 (34.3)	1,526 (41.3)	1,560 (52.2)	1,070 (63.7)
Liver disease, (%)	3,050 (44.6)	1,855 (50.2)	1,074 (36.0)	690 (41.1)
**Hospital outcomes**				
Inpatient mortality, (%)	96 (1.4)	82 (2.2)	94 (3.1)	124 (7.4)
Length of stay, mean (SD), d	5.0 (8.6)	4.7 (5.6)	6.1 (10.9)	6.8 (9.3)
ICU admission, (%)	2,042 (29.9)	1,057 (28.6)	765 (25.6)	523 (31.1)
Readmission within 30 days, (%)	1,211 (18.0)	580 (16.0)	577 (19.9)	345 (22.2)

[a]*Includes any benzodiazepine, clonidine, dexmedetomidine, gabapentin, phenobarbital, and/or valproic acid.*
[b]*Includes only the 5 most common comorbidities.*
[c]*Includes complicated and uncomplicated hypertension.*
Abbreviations: AWS, alcohol withdrawal; CIWA-Ar, Clinical Institute Withdrawal Assessment for Alcohol, revised;[8] COPS2, Comorbidity Point Score, version 2;[29] ICU, intensive care unit; IQR, interquartile range; LAPS2, Laboratory Acute Physiology Score, version 2;[29] SD, standard deviation.

© 2022 Smith JT et al. *JAMA Network Open.*

eTable 5. Baseline Characteristics of Hospitalizations for Alcohol Withdrawal Syndrome With Use ≥1 Benzodiazepine[a]

	With use of an orderset		Without use of an orderset	
**Characteristic**	**Pre-implementation (n = 6,431)**	**Post-implementation (n = 2,823)**	**Pre-implementation (n = 2,114)**	**Post-implementation (n = 920)**
Age, mean (SD), years	52.6 (13.3)	52.7 (13.8)	57.9 (14.0)	58.8 (14.8)
Male, (%)	4,488 (69.8)	1,943 (68.8)	1,372 (64.9)	575 (62.5)
Observation admission, (%)	668 (10.4)	231 (8.2)	144 (6.8)	25 (2.7)
Weekend admission, (%)	1,686 (26.2)	750 (26.6)	354 (16.7)	192 (20.9)
LAPS2, mean (SD)	68.8 (37.2)	76.3 (39.9)	51.4 (44.5)	76.9 (49.1)
COPS2, mean (SD)	39.7 (43.1)	40.3 (46.3)	53.1 (51.9)	59.7 (60.5)
Urine toxicology checked (%)	2,842 (44.2)	1,443 (51.1)	393 (18.6)	260 (28.3)
Had ≥1 prior hospitalization for AWS within prior 6 months, (%)	2,238 (34.8)	1,058 (37.5)	437 (20.7)	185 (20.1)
Had ≥1 CIWA-Ar value recorded, (%)	6,401 (99.5)	2,776 (98.3)	396 (18.7)	66 (7.2)
**Elixhauser comorbidities[b]**				
Alcohol abuse, (%)	5,111 (79.5)	2,393 (84.8)	2,016 (95.4)	905 (98.4)
Hypertension[c], (%)	3,684 (57.3)	1,656 (58.7)	1,323 (62.6)	607 (66.0)
Fluid and electrolyte disorders, (%)	3,979 (61.9)	1,862 (66.0)	956 (45.2)	561 (61.0)
Peripheral vascular disease, (%)	2,126 (33.1)	1,096 (38.8)	1,046 (49.5)	571 (62.1)
Liver disease, (%)	2,883 (44.8)	1,452 (51.4)	767 (36.3)	373 (40.5)
**Hospital outcomes**				
Inpatient mortality, (%)	92 (1.4)	74 (2.6)	77 (3.6)	97 (10.5)
Length of stay, mean (SD), d	5.1 (8.7)	4.9 (5.9)	6.4 (11.8)	8.1 (11.1)
ICU admission, (%)	1,939 (30.2)	872 (30.9)	572 (27.1)	338 (36.7)
Readmission within 30 days, (%)	1,134 (17.9)	445 (16.2)	383 (18.8)	178 (21.6)

[a]*Includes alprazolam, chlordiazepoxide, diazepam, lorazepam, midazolam, oxazepam, and/or temazepam.*
[b]*Includes only the 5 most common comorbidities.*
[c]*Includes complicated and uncomplicated hypertension.*
Abbreviations: AWS, alcohol withdrawal; CIWA-Ar, Clinical Institute Withdrawal Assessment for Alcohol, revised;[8] COPS2, Comorbidity Point Score, version 2;[29] ICU, intensive care unit; IQR, interquartile range; LAPS2, Laboratory Acute Physiology Score, version 2;[29] SD, standard deviation.

© 2022 Smith JT et al. *JAMA Network Open.*

eTable 6. Baseline Characteristics of Hospitalizations for Alcohol Withdrawal Syndrome 12 mo Before and After Implementation

	With use of an orderset		Without use of an orderset	
**Characteristic**	**Pre-implementation (n = 2,568)**	**Post-implementation (n = 4,652)**	**Pre-implementation (n = 1,881)**	**Post-implementation (n = 3,709)**
Age, mean (SD), years	53.6 (14.2)	54.7 (14.7)	59.4 (15.0)	61.7 (15.3)
Male, (%)	1,775 (69.1)	3,244 (69.7)	1,309 (69.6)	2,475 (66.7)
Observation admission, (%)	246 (9.6)	327 (7.0)	114 (6.1)	94 (2.5)
Weekend admission, (%)	699 (27.2)	1,239 (26.6)	359 (19.1)	795 (21.4)
LAPS2, mean (SD)	67.8 (37.7)	73.8 (39.7)	54.5 (43.6)	69.4 (45.8)
COPS2, mean (SD)	39.8 (43.6)	40.4 (46.9)	60.3 (58.0)	63.1 (62.0)
Urine toxicology checked (%)	1,130 (44.0)	2,129 (45.8)	325 (17.3)	777 (20.9)
Had ≥1 prior hospitalization for AWS within prior 6 months, (%)	822 (32.0)	1,545 (33.2)	364 (19.4)	719 (19.4)
Had ≥1 CIWA-Ar value recorded, (%)	2,543 (99.0)	4,525 (97.3)	210 (11.2)	159 (4.3)
**Elixhauser comorbidities[a]**				
Alcohol abuse, (%)	2,164 (84.3)	4,145 (89.1)	1,816 (96.5)	3,654 (98.5)
Hypertension[b], (%)	1,501 (58.5)	2,814 (60.5)	1,232 (65.5)	2,555 (68.9)
Fluid and electrolyte disorders, (%)	1,548 (60.3)	2,942 (63.2)	844 (44.9)	2,086 (56.2)
Peripheral vascular disease, (%)	935 (36.4)	2,006 (43.1)	990 (52.6)	2,335 (63.0)
Liver disease, (%)	1,110 (43.2)	2,229 (47.9)	669 (35.5)	1,515 (40.8)
**Hospital outcomes**				
Inpatient mortality, (%)	41 (1.6)	93 (2.0)	32 (1.7)	166 (4.5)
Length of stay, mean (SD), d	4.4 (6.8)	4.4 (5.4)	4.6 (6.6)	5.2 (7.0)
ICU admission, (%)	641 (25.0)	1,229 (26.4)	397 (21.1)	841 (22.7)
Readmission within 30 days, (%)	451 (17.8)	718 (15.7)	392 (21.2)	766 (21.6)

[a] *Includes only the 5 most common comorbidities.*
[b] *Includes complicated and uncomplicated hypertension.*
Abbreviations: AWS, alcohol withdrawal; CIWA-Ar, Clinical Institute Withdrawal Assessment for Alcohol, revised;[8] COPS2, Comorbidity Point Score, version 2;[29] ICU, intensive care unit; IQR, interquartile range; LAPS2, Laboratory Acute Physiology Score, version 2;[29] SD, standard deviation.

© 2022 Smith JT et al. *JAMA Network Open.*

eMethods. SAS Code for Difference-in-Differences Analysis

```
%macro did(outcome,dist,link,where);
PROC GEE DATA = etohrx;
&where.;
class post mrn orig_fac_id os mon;
model &outcome.(event='1')=age_at_admit male cops2 laps2 prior_hosp obs_only admit_weekend mon
urinetox post os os*post time0 time1 os*time0 os*time1 orig_fac_id/dist=&dist. link=&link.;
REPEATED subject = MRN/ type=exch covb corrw;
lsmeans os*post/cl ilink;
estimate os*post 1 -1 -1 1/cl ilink;
output out=adj pred=p0;
ods output GEEEmpPEst=geeest lsmeans=lsm estimates=diff parameterestimates=beta;
run;

data beta;
set beta;
outcome="&outcome.";
if parameter eq 'time0*os' and level1 eq 'No';
if "&link." eq "identity" and outcome eq 'los_day' then outcome='los_linear';
if "&link." eq "identity" and outcome eq 'lorazepam_tot' then outcome='lor_linear';
run;

data betas;
        set betas beta;
        run;

        data diff;
        set diff;
        outcome="&outcome.";
        if "&link." eq "identity" and outcome eq 'los_day' then outcome='los_linear';
        if "&link." eq "identity" and outcome eq 'lorazepam_tot0' then outcome='lor_linear';
        run;

        data lsme;
        set lsm;
        outcome="&outcome.";
        if "&link." eq "identity" and outcome eq 'los_day' then outcome='los_linear';
        if "&link." eq "identity" and outcome eq 'lorazepam_tot0' then outcome='lor_linear';
        run;

        data lsmes;
        set lsmes lsme;
        run;

        data diffs;
        set diffs diff;
        run;
        %mend;
```

Legend: &where, used to remove deaths for the readmission outcome; &link, LOG link for POISSON and GAMMA distributions and IDENTITY for normal; &dist, GAMMA and NORMAL for length of stay and POISSON for the binary outcomes; Time0, denotes the month number starting with month=1 until the end of the study; Time1, set to 0 for months in the pre-period and has values 1 to number of months in the post period.

© 2022 Smith JT et al. *JAMA Network Open.*
